# Uveodermatological syndrome in dogs: A review of diagnosis, management, and ophthalmic patient needs

**DOI:** 10.17221/10/2025-VETMED

**Published:** 2025-06-25

**Authors:** Pavol Zubricky, Agnieszka Balicka, Zuzana Drahovska, Maria Lapsanska, Alexandra Trbolova

**Affiliations:** Small Animal Clinic, University of Veterinary Medicine and Pharmacy in Kosice, Slovak Republic

**Keywords:** autoimmune diseases, canine, depigmentation, uveitis

## Abstract

Uveodermatological syndrome is a widely recognised disease that continues to raise significant concern among both veterinarians and pet owners. Its aetiology, although still unclear, is believed to involve an autoimmune origin and genetic predisposition. The most common clinical signs include skin depigmentation, alopecia, poliosis, and, most frequently, granulomatous panuveitis. Affected dogs often develop secondary complications owing to persistent intraocular inflammation, such as cataracts and glaucoma. Although immunosuppressive therapy often yields a favourable response, recurrence is commonly observed. In some cases, uveodermatological syndrome presents a clinical challenge, as patients may experience adverse effects from medications, without which irreversible blindness may ensue.

## INTRODUCTION

In humans, Vogt–Koyanagi–Harada syndrome (VKH), also known as uveomeningoencephalitic syndrome, is a multisystemic autoimmune disease characterised by ophthalmic, dermatologic, and neurologic clinical signs (Gaudreau et al. 2012). In veterinary medicine, VKH is considered analogous to its human counterpart, differing primarily in the absence or rare occurrence of neurological signs. Although the exact cause of uveodermatological syndrome remains unclear, immune-mediated reactions targeting melanocytes have been proposed as a potential underlying mechanism (Yamaki et al. 2000; Angles et al. 2005).

The syndrome was first described by two scientists, Vogt and Koyanagi, and the name was derived from their surnames. In the 1950s, the name was modified with the addition of Harada, who was one of the first to thoroughly characterise the disease, leading to the current designation of Vogt–Koyanagi–Harada (VKH) syndrome (O’Keefe and Rao 2017). To this day, numerous publications – likely owing to convention – refer to VKH as a “syndrome” rather than a “disease”, despite its classification as a “disease” by the International Committee on Nomenclature in 2001 (Read et al. 2001).

The first similarities between the disease in humans and animals were observed in 1977 in Japan, specifically in the Akita Inu dog (Asakura et al. 1977). In 1985, Romanowski introduced the term “uveodermatological syndrome (UDS)” into the veterinary literature (Romanowski 1985), arguing against the use of the term “canine VKH syndrome” owing to the absence of meningeal involvement. In current veterinary practice, the terms UDS and VKH or VKH-like syndrome are often used interchangeably.

## MATERIAL AND METHODS

This review is based on the collection of published articles concerning “uveodermatological syndrome in dogs”. A literature search was conducted in the PubMed, Web of Science, ScienceDirect, and Google Scholar databases using the following keywords, either in combination or separately: “uveodermatological syndrome”, “Vogt–Koyanagi–Harada-like syndrome”, “UVD”, and “VKH” in dogs. The research focused on descriptions of affected canine patients presenting with ophthalmological issues, including clinical signs, therapy, and the general disease course. Literature research was conducted between November 2024 and January 2025. All articles cited in this review are appropriately referenced in the bibliography. We selected literature that provided the most relevant and current information on our search terms within veterinary medicine, excluding data that focused exclusively on dermatological aspects of the disease. This review aims to provide accessible information regarding the ocular manifestations of uveodermatological syndrome in dogs, to analyse case reports, aetiology, diagnostics, and therapeutic approaches recognised in the current literature.

## AETIOLOGY

Uveodermatological syndrome in certain breeds, including Akita Inu, Samoyed, and Siberian Husky, is considered an ocular disorder with both hereditary and immune-mediated components. Although the genetic background has been confirmed, no comprehensive commercial genetic tests are currently available for dogs.

Similar to VKH in humans, an immune-mediated reaction against melanocytes is believed to be responsible for UDS. However, the immunologic basis of this inflammation remains to be fully elucidated (Kang et al. 2014). Melanocytes possess the unique ability to synthesise melanin pigments, which determine skin and hair colour in both humans and animals. In domesticated mammals, melanocytes are present not only in the skin and its appendages but also in the oral mucosa, eye, cochlea, and meninges (Tham et al. 2019). In the eye, melanocytes are abundant in the uvea, which includes the iris, ciliary body, and choroid (Tham et al. 2019). The uveal pigment is thought to shield the retina from overexposure to solar radiation and its damaging effects (Pandiani et al. 2017).

Immunohistochemical studies in humans have demonstrated that approximately 70% of lesional lymphocytes are T cells (Inomata and Sakamoto 1990). Further analysis has revealed that the choroidal infiltrate consists predominantly of helper T cells (Sakamoto 1991). Moreover, it has been shown that in humans with UDS, tyrosinase peptide antigens are the targets of autoimmune T cells. Overall, UDS is considered an autoimmune disease in which cell-mediated immunity plays a central role in its pathogenesis (Tham et al. 2019).

In both humans and dogs, UDS is thought to result from a Th1 lymphocyte-driven, cell-mediated immune response, leading to autoimmune attacks on melanocytes in the skin, uvea, and, in humans, the CNS and inner ear (Sigle et al. 2006). Although the pathogenesis of UDS in dogs remains incompletely understood, experimental immunisation of two Akita dogs with tyrosinase-related protein resulted in clinical and histological features consistent with UDS. Tyrosinase proteins are enzymes involved in melanin synthesis and are expressed specifically in melanocytes (Yamaki et al. 2000). In addition, studies have demonstrated that the canine leukocyte antigen DLA-DQA1*00201 is associated with a significantly increased relative risk for UDS in American Akitas compared to other DLA Class II alleles (Angles et al. 2005).

In dogs, an enrichment of T cell gene signatures has also been documented, with upregulation of *IFNG*, *TNF*, *PRF1*, *IL15*, *CTSW*, *CXCL10*, and *CCL5* in dogs affected by VKH and vitiligo compared to unaffected controls. Similar findings have been reported in humans, suggesting these genes may contribute to the spontaneous pathogenesis of VKH and vitiligo. Additional studies have identified enrichment of T cell-associated genes such as *FOXP3* and *TBX21*, with concurrent downregulation of *IGFBP5*, *FOXO1*, and *PECAM1*. Furthermore, *TGFB3*, *SFRP2*, and *CXCL7* have been proposed as additional drivers of autoimmune pigmentary disorders (Egbeto et al. 2020).

Although the viral infection hypothesis has been proposed as a potential trigger in humans, the role of antiretinal antibodies (ARAs) remains unclear. The viral aetiology remains controversial, as autoreactivity to retinal proteins appears to vary between acute and chronic phases of the disease. Additionally, antibodies may be produced secondarily to retinal damage (Lavezzo et al. 2016; Tham et al. 2019).

A gene expression study in dogs affected with UDS concluded that further research is essential to better understand the immunopathogenesis of spontaneous autoimmunity. Such knowledge is critical for the development of targeted therapies in both veterinary and human medicine and may aid in predicting disease prognosis and treatment response (Egbeto et al. 2020).

## CLINICAL SIGNS

Uveodermatological syndrome has been reported in various breeds and mixed-breed dogs, most commonly in Akitas (66%), as well as in Siberian Huskies, Alaskan Malamutes, Samoyeds, Irish Setters, Basset Hounds, Golden Retrievers, Shetland Sheepdogs, Old English Sheepdogs, Saint Bernards, Chow Chows, Dachshunds, Fox Terriers, Jack Russell Terriers, Rat Terriers, Brazilian Fila dogs, Bernese Mountain Dogs, and Miniature Poodles (Sarna 1992; Scott 2001; Pandiani et al. 2017; Tham et al. 2019). To our knowledge, UDS has not been reported in cats or horses.

Although retrospective studies in human medicine suggest a higher prevalence in North America, there is no evidence of regional predisposition in dogs. Canine cases of UDS have been documented globally, including in Asia, Europe, South America, and North America (Tham et al. 2019).

While the sequence of symptoms may vary, ocular signs often precede dermatological manifestations (Kang et al. 2014). In some cases, owners first notice progressive vision loss (Thomas and Chahory 2009). Dermatological signs of UDS include poliosis, multiple areas of alopecia, and progressive skin depigmentation on the scrotum, nasal planum, and mucocutaneous junctions of the mouth, as well as the perianal and periocular regions (Kern et al. 1985; Sigle et al. 2006; Kang et al. 2013; Gelatt 2021). In certain instances, ulcerated skin with crusts around the eyes may also be observed (Thomas and Chahory 2009).

**Figure 1:**
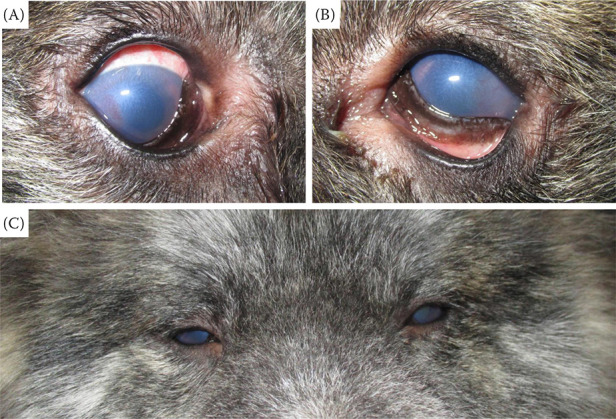
Female, 4-year-old, mixed breed dog affected with uveodermatological syndrome (A) Right eye with corneal oedema, neovascularisation, and dyscoria owing to posterior synechiae; (B) Left eye with corneal oedema, neovascularisation, and iris bombé; (C) Blepharospasm of the affected eyes

**Figure 2:**
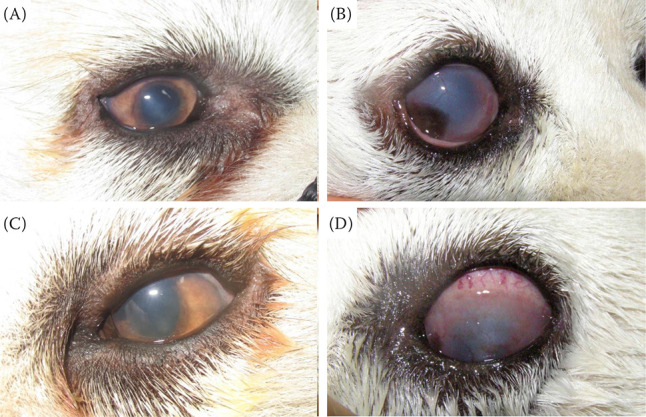
Male, 3-year-old, Akita Inu dog affected with Uveodermatological syndrome Right eye (A) and left eye (C) during the first examination. Visible corneal oedema, aqueous flare, rubeosis iridis, and iris bombe. Right eye (B) and left eye (D), 18 months after the first examination. The patient was presented owing to deterioration and relapse of the disease. Secondary glaucoma caused blindness, corneal oedema, pigmentation, and neovascularisation, enabling examination of the anterior chamber

The ophthalmic signs of UDS are numerous and may include ocular discharge, blepharospasm, photophobia, diminished or absent pupillary light reflex (PLR), conjunctival hyperemia, third eyelid swelling, congestion of episcleral vessels, anterior uveitis, hyphema, keratic precipitates, aqueous flare, rubeosis iridis or iris depigmentation, posterior synechiae, chorioretinitis, and occasionally retinal detachment (Barros et al. 1991; Laus et al. 2004; Kang et al. 2013) (Figures 1 and 2). Glaucoma, cataracts, and iris bombé are regarded as secondary changes resulting from chronic inflammation (Laus et al. 2004). A comparison of individual cases described in the literature featuring ophthalmologic symptoms of UVD is presented in Table 1.

**Table 1:** Initial clinical presentation and therapeutic interventions in affected dogs

Patient description	First clinical signs observed by the owner	Clinical signs	Therapy	Course of the disease	References
5-year-old, male Brazilian Fila dog	vision loss associated with alopecia	- menace: decreased - pupillary light reflexes: decreased - severe conjunctival hyperaemia, and congestion of the episcleral vessels - diffuse corneal oedema - keratic precipitates, iridal oedema, rubeosis iridis or annular posterior synechiae, with beginning of iris bombé - hyphema in the left eye - ophthalmoscopy did not show fundic alterations - IOP OD 7, OS 2 mmHg	- **oral medications:** prednisone - **subconjunctival injection:** methylprednisone - **topical medications:** prednisone, indometacine, dorzolamide with timolol	successful, the patient remains on therapy	Laus et al. (2004)
7-year-old, Siberian Husky-type dog, spayed female Heterochromia irides was apparent, the dog had a brown iris in the right eye and a blue iris in the left eye	3-day history of blepharospasm, photophobia, and serous ocular discharge from the right eye	- menace response: absent - dazzle reflex: present - direct pupillary light reflex: absent in the right eye but present in the left eye - indirect pupillary light reflexes: absent in both eyes - episcleral injection; conjunctival hyperaemia; and mild, diffuse corneal oedema in the right eye - pronounced aqueous flare with diffuse iris thickening, rubeosis iridis, iris bombé associated with posterior synechiae precluded - complete evaluation of the lens of the right eye - cupping of the optic disk (with a small area of haemorrhage over the optic disk); generalised attenuation of the retinal vasculature; and multifocal, poorly demarcated depigmented lesions in the nontapetal fundus (subalbinotic tapetal fundus of the left eye was considered normal in appearance) - IOP OD 53, OS 14 mmHg	- **oral medications:** prednisone - **topical medications:** prednisolone, atropine, brinzolamide	enucleation of the right eye	Sigle et al. (2006)
7-year-old, Siberian Husky-type Dog, spayed female Heterochromia irides was apparent, the dog had a brown iris in the right eye and a blue iris in the left eye	3-day history of blepharospasm, photophobia, and serous ocular discharge from the right eye	- menace response: absent - dazzle reflex: present - direct pupillary light reflex: absent in the right eye but present in the left eye - indirect pupillary light reflexes: absent in both eyes - episcleral injection; conjunctival hyperaemia; and mild, diffuse corneal oedema in the right eye - pronounced aqueous flare with diffuse iris thickening, rubeosis iridis, iris bombé associated with posterior synechiae precluded - complete evaluation of the lens of the right eye - cupping of the optic disk (with a small area of haemorrhage over the optic disk); generalised attenuation of the retinal vasculature; and multifocal, poorly demarcated depigmented lesions in the nontapetal fundus (subalbinotic tapetal fundus of the left eye was considered normal in appearance) - IOP OD 53, OS 14 mmHg	- **oral medications:** prednisone - **topical medications:** prednisolone, atropine, brinzolamide	enucleation of the right eye	Sigle et al. (2006)
8-year-old, spayed female Japanese Akita	acute blindness, cloudy eyes, and squinting	- menace response: absent - direct pupillary light reflex: severely decreased in both eyes - dazzle reflex: absent on left eye, decreased on right eye - blepharospasm bilaterally - bilateral moderate aqueous flare, iris hyperaemia, and keratic precipitates - bilateral bullous retinal detachment - accompanied by a clear fluid underlying the retina seen in both eyes - neuro-ophthalmic examination: normal - IOP OD 12, OS 6 mmHg	- **oral medications:** azathioprine, prednisone - **topical medications:** prednisolone	Complete restoration of vision	Pye (2009)
1-year old, male Jack Russell Terrier First reported case of polymyositis subsequent to VKH-like disease in a dog	bilateral uveitis and glaucoma	- episcleral congestion - miosis, markedly narrowed anterior chambers, aqueous flare - corneal and perilimbal oedema - USG: retinal detachment in the right eye - IOP OD 44, OS 40 mmHg	no information	- right eye: enucleated (left eye enucleated owing to lack of improvement 3 months after therapy) - euthanasia 3 years later	Baiker et al. (2011)
4-year-old, male Rat Terrier	severe bilateral nonresponsive panuveitis	- blepharospasm, episcleral injection, and moderate conjunctival hyperaemia - menace, tracking test, dazzle reflex: absent - both direct and consensual pupillary light reflexes: absent - pupils were miotic with mild to moderate swelling of the iris base - diffuse corneal oedema, aqueous flare with cells, and fibrin adhered to the anterior lens capsules bilaterally - peripheral bullous retinal detachment with focal perivascular infiltrates and haemorrhages OU - IOP OD 48, OS 36 mmHg	- **oral medications:** doxycycline, prednisone, famoti-dine - **topical medications:** prednisolone, dorzolamide, timolol, atropine	patient remains on therapy	Blackwood et al. (2011)
2-year-old, intact male Siberian Husky	Conjunctivitis and uveitis	- menace response, dazzle response, direct and consensual pupillary light reflexes: absent - blepharospasm, conjunctival hyperemia - diffuse corneal oedema - third eyelid swelling and prolapsed - USG: anterior chamber shallow, marked opacification of lenses in both eyes, posterior globe wall: thickened, amorphous opacities seen in the vitreous - IOP OD 8, OS 6 mmHg	no information	dog was euthanised	Kang et al. (2013)
5-year-old, intact female Miniature Poodle dog	history of "red" eye with radual blindness	- menace, dazzle response, and visual placing: absent - direct and consensual pupillary light reflexes: absent - blepharospasm, conjunctival hyperemia, diffuse corneal oedema with severe vascularisation, and mucopurulent discharge - anterior and posterior uvea, lens and fundus not visible (because of severe corneal oedema and vascularisation) - neuro-ophthalmic examination: normal - IOP OD 11, OS 12 mmHg	- **oral medications:** prednisolone - **topical medications:** cyclosporine, sodium hyaluronate	uveitis continued to progress	Kang et al. (2014)

IOP = intraocular pressure; OD = right eye; OS = left eye

Additionally, uveodermatological syndrome concurrent with keratoconjunctivitis sicca has been described in a 5-year-old female Miniature Poodle (Tham et al. 2019). Another patient, a 7-year-old Siberian Husky-type dog with unilateral uveitis diagnosed with UDS, showed a reduced Schirmer Tear Test, although no corneal or conjunctival changes were observed. The reduced tear production is considered to result from immune-mediated lacrimal adenitis (Sigle et al. 2006).

The severity of ocular lesions varies between individual cases, ranging from bilateral anterior uveitis to panuveitis. As progressive depigmentation of the iris and retinal pigment epithelium occurs, the tapetal fundus becomes hyperreflective, and retinal vessels are attenuated. Over time, optic nerve atrophy may develop, and bullous retinal detachments may occur (Gelatt 2021). In dogs affected by secondary glaucoma, optic nerve cupping and haemorrhage over the optic disc have been described (Sigle et al. 2006).

No sex predisposition has been observed in animals (in contrast to human medicine, where women are more often affected), although roughly two-thirds of affected dogs were male in one retrospective study (Zarfoss et al. 2018). The age of affected animals ranges from 6 months to 6 years (Barros et al. 1991). In most described cases, clinical signs are bilateral, although one unilateral case has been reported in a 7-year-old Siberian Husky with *heterochromia iridium*. This dog presented with facial and truncal poliosis, vitiligo, and right-eye uveitis. Uveitis was observed in the brown eye, while the blue eye was unaffected. In this dog, tear production was decreased in both eyes. Owing to the presumed pathophysiology of UDS, the likelihood of unilateral uveitis is thought to be greater in dogs with *heterochromia iridium*, than in dogs with bilateral brown eyes (Thomas and Chahory 2009).

Differential diagnoses of dermatologic lesions should include cutaneous lymphoma, vitiligo, discoid lupus erythematosus, pemphigus foliaceus, and pemphigus erythematosus (Kang et al. 2014). In most described cases, the complete blood count, serum chemistry profile, and thyroid function tests are normal in UDS-affected dogs (Kang et al. 2013; Zarfoss et al. 2018).

In the case of UDS, there are no specific diagnostic criteria available in the literature. The diagnosis is based on the history, ophthalmic examination results, and histological findings of skin lesions (Kang et al. 2014). To our knowledge, only two reported cases of neurologic signs in UDS-affected dogs exist in the literature (Cottell and Barnett 1987; Denerolle et al. 2000).

## HISTOPATHOLOGY

In patients where UDS is suspected, skin biopsies are indispensable for confirming the diagnosis. Early diagnosis is crucial owing to the need for prompt therapy to reduce the risk of blindness. The diagnostic procedure is based on multiple skin biopsies collected from lesions or their margins, including depigmented areas, particularly those with active inflammation, erythema, or swelling (Tham et al. 2019). Biopsy results in dogs with UDS reveal histiocytic lichenoid dermatitis with an absence of pigment within keratinocytes. The inflammatory cell composition includes histiocytes, lymphocytes, and melanophages. Additionally, Schmorl’s staining reveals the absence of epidermal melanocytes and pigmentary incontinence, with scattered melanin granules present in macrophages (Laus et al. 2004; Kang et al. 2013).

Histopathological examination of the enucleated globes of a 2-year-old Siberian Husky affected by UDS revealed diffuse pyogranulomatous inflammation of the iris, ciliary body, retina, and choroid, confirming both anterior and posterior uveitis with bilateral degeneration and bullous retinal detachment. Additionally, lymphocytic and histiocytic inflammation with several macrophages containing melanin granules in the cytoplasm was present (Kang et al. 2013).

Granulomatous inflammation centred on pigmented cells is a defining feature of canine UDS. Clusters of histiocytes, in a manner reminiscent of Dalen-Fuchs nodules, can be observed within the choroid beneath the retinal pigment epithelium (RPE). These collections of epithelioid cells between the RPE and Bruch’s membrane are associated with granulomatous uveitis, including Vogt–Koyanagi–Harada (VKH) disease in humans (Dubielzig et al. 2010).

Although bacteriological examination of affected skin samples is negative in most cases, cytological examination may reveal secondary infection with cocci (Kang et al. 2013).

## THERAPY

Although UDS is not a life-threatening disease, it is difficult to control (Thomas and Chahory 2009). Despite intensive long-term systemic immunosuppressive therapy and early diagnosis, permanent vision loss often occurs in affected dogs (Lindley et al. 1990; Baiker et al. 2011; Blackwood et al. 2011). The main goal of local and systemic therapy is to reduce inflammation using immunosuppressive and anti-inflammatory drugs and to prolong the time before the onset of blindness (Zarfoss et al. 2018). In dogs with ocular signs, UDS therapy focuses on stabilising intraocular inflammation, managing pain, and addressing secondary complications such as glaucoma.

Topical corticosteroids inhibit chemotaxis and neovascularisation, reduce cellular and protein exudation, and stabilise lysosomal membranes. They block the conversion of membrane phospholipids to arachidonic acid, preventing activation of pro-inflammatory cyclooxygenase and lipoxygenase pathways. Several topical corticosteroids are available, varying in potency and corneal penetration. Prednisolone acetate, owing to its excellent corneal penetration, is widely recommended for anterior uveitis. Dexamethasone also penetrates the cornea effectively, though less so than prednisolone, and is typically used for ocular surface therapy. Less potent corticosteroids, such as hydrocortisone, are inadequate for intraocular conditions. Long-term topical corticosteroid use may cause adverse effects, including subcapsular cataracts and corneal lipidosis. Systemic effects, such as polyuria and polydipsia, may also occur (Gould and McLellan 2014).

In addition to corticosteroids, ciclosporin and azathioprine are immunomodulatory agents used via various formulations. These drugs bind to intracytosolic immunophilins in T helper cells and block lymphokine production necessary for T cell activation. Topical ciclosporin is used for chronic keratitis and dry eye. In corticosteroid-resistant UDS cases, azathioprine is often the preferred alternative, used alone or in combination. Owing to possible side effects – including hepatotoxicity, bone marrow suppression, and gastrointestinal disorders – azathioprine requires careful monitoring (Gould and McLellan 2014).

Prednisolone is recommended at doses above 1 mg/kg per day, tapered gradually. A review of 50 UDS cases reported doses ranging from 0.26 mg/kg to 2 mg/kg, depending on individual cases (Zarfoss et al. 2018). Corticosteroids suppress Th1 (and indirectly Th2) responses and inhibit macrophage activation. Relapse is common, and recurrences may be harder to control. Azathioprine, active against Th1 responses, is commonly combined with corticosteroids at 2 mg/kg/day. Owners must be informed that the response to azathioprine may take up to 6 weeks (Thomas and Chahory 2009). In some cases, cyclosporine A may replace azathioprine, although cost may be a limiting factor (Thomas and Chahory 2009).

Topical prednisolone drops every 4–6 h are recommended for intraocular inflammation. Adjunct therapy may include anti-glaucoma agents (e.g., brinzolamide, brinzolamide/timolol) and atropine to break down synechiae. Atropine should be used cautiously due to its potential to reduce tear production and affect IOP (Sigle et al. 2006). Some authors report success with 1% cyclosporine applied topically twice daily with prednisolone (Gould and McLellan 2014).

A 5-year-old Toy Poodle with UDS and KCS received sodium hyaluronate every 4 hours. After failing to respond to systemic prednisolone after 3 weeks, mycophenolate mofetil was administered orally at 10 mg/kg every 8 hours (Kang et al. 2014). In another case, two subconjunctival injections of 8 mg methylprednisolone, 15 days apart, were used. Despite aggressive treatment, some dogs show insufficient improvement. In a miniature poodle, bilateral enucleation was performed owing to persistent inflammation. The skin lesions improved, but the owners declined further treatment (Kang et al. 2014). Enucleation may be recommended in blind dogs with persistent inflammation or drug side effects to improve quality of life.

Although recovery of visual function may occur, it often declines with prednisolone tapering (Zarfoss et al. 2018). In several cases, tapering is necessary owing to adverse effects. In a retrospective study of 42 UDS dogs treated with prednisone, 36% experienced side effects necessitating dose reduction or discontinuation. Side effects included polyuria, polydipsia, hepatopathy, muscle wasting, diarrhoea, lethargy, urinary tract infections, anorexia, inappropriate urination, and polyphagia (Zarfoss et al. 2018).

Lack of improvement in skin or ocular signs, or the development of new or progressing lesions, is considered treatment failure. In human medicine, early high-dose corticosteroids are critical. In veterinary medicine, no standardised protocol exists, and therapy duration varies. Better outcomes are reported when treatment begins within one month of symptom onset. There are no reports of spontaneous remission (Tham et al. 2019).

## CONCLUSION

Although UDS is continually studied in human and veterinary medicine, its aetiology remains poorly understood. It presumably has both hereditary and immune-mediated components. Management of individual cases must be based on interdisciplinary collaboration between an ophthalmologist and a dermatologist to control the disease’s dermatological and ocular signs.

In some cases, UDS is difficult to control, and relapses are frequent. Chronic intraocular inflammation often leads to permanent blindness in the affected eye.
